# MERS-CoV and SARS-CoV-2 replication can be inhibited by targeting the interaction between the viral spike protein and the nucleocapsid protein

**DOI:** 10.7150/thno.55647

**Published:** 2021-02-06

**Authors:** Byoung Kwon Park, Jinsoo Kim, Sangkyu Park, Dongbum Kim, Minyoung Kim, Kyeongbin Baek, Joon-Yong Bae, Man-Seong Park, Won-Keun Kim, Younghee Lee, Hyung-Joo Kwon

**Affiliations:** 1 Institute of Medical Science, College of Medicine, Hallym University, Chuncheon 24252, Republic of Korea.; 2 Department of Microbiology, College of Medicine, Hallym University, Chuncheon 24252, Republic of Korea.; 3 Department of Biochemistry, College of Natural Sciences, Chungbuk National University, Cheongju 28644, Republic of Korea.; 4 Department of Microbiology, College of Medicine, and the Institute for Viral Diseases, Korea University, Seoul 02841, Republic of Korea.

**Keywords:** MERS-CoV, SARS-CoV-2, nucleocapsid protein, spike protein, targeting

## Abstract

**Background:** The molecular interactions between viral proteins form the basis of virus production and can be used to develop strategies against virus infection. The interactions of the envelope proteins and the viral RNA-binding nucleocapsid (N) protein are essential for the assembly of coronaviruses including the Middle East respiratory syndrome coronavirus (MERS-CoV).

**Methods:** Using co-immunoprecipitation, immunostaining, and proteomics analysis, we identified a protein interacting with the spike (S) protein in the cells infected with MERS-CoV or SARS-CoV-2. To confirm the interaction, synthetic peptides corresponding to the C-terminal domain of the S protein (Spike CD) were produced and their effect on the interaction was investigated *in vitro*. *In vivo* effect of the Spike CD peptides after cell penetration was further investigated using viral plaque formation assay. Phylogeographic analyses were conducted to deduce homology of Spike CDs and N proteins.

**Results:** We identified a direct interaction between the S protein and the N protein of MERS-CoV that takes place during virus assembly in infected cells. Spike CD peptides of MERS-CoV inhibited the interaction between the S and N proteins *in vitro.* Furthermore, cell penetration by the synthetic Spike CD peptides inhibited viral plaque formation in MERS-CoV-infected cells. Phylogeographic analyses of Spike CDs and N proteins showed high homology among betacoronavirus lineage C strains. To determine if Spike CD peptides can inhibit the replication of severe acute respiratory syndrome coronavirus 2 (SARS-CoV-2), we used the same strategy and found that the SARS-CoV-2 Spike CD peptide inhibited virus replication in SARS-CoV-2-infected cells.

**Conclusions:** We suggest that the interaction between the S protein and the N protein can be targeted to design new therapeutics against emerging coronaviruses, including SARS-CoV-2.

## Introduction

Middle East respiratory syndrome coronavirus (MERS-CoV) causes acute respiratory infection and has been transmitted around the world since the first report in Saudi Arabia in June 2012 1-3. MERS-CoV generally transmits between dromedary camels, from dromedary camels to humans, or between humans 1-4. A total of 2,494 people in 27 countries have been infected with MERS-CoV since September 2012, of which 858 have died, resulting in a mortality rate around 34% (http://www.who.int/emergencies/mers-cov/en/). In South Korea, in 2015, there were 186 laboratory-confirmed cases of MERS-CoV infection, resulting in 38 fatalities 5. Most of the MERS-CoV infections (92.5%) in South Korea occurred within hospitals, with five super-spreaders responsible for 83% of the total events 6.

MERS-CoV is a single-stranded RNA virus that expresses several proteins, including envelope proteins [spike (S), membrane (M), and envelope (E)], a nucleocapsid (N) protein, and accessory proteins (3, 4a, 4b, 5, and 8b) 7-9. The envelope proteins play an important role in viral entry into cells. First, the receptor-binding domain (RBD) of the S1 region of the S protein attaches to the human dipeptidyl peptidase 4 (hDPP4) receptor on a host cell. Then, the fusion peptide region is inserted into the host-cell membrane after formation of an antiparallel six-helix bundle based on the heptad repeats domain 10-12. Because of its vital role in host-cell entry, the S protein has gained attention as a target for diagnostic reagents and therapeutic antibodies against MERS-CoV infection 13.

Coronaviruses (CoVs) such as mouse hepatitis virus (MHV) and severe acute respiratory syndrome coronavirus (SARS-CoV) assemble in the endoplasmic reticulum-Golgi intermediate compartment (ERGIC) 14, 15. After the viral envelope proteins are translated, they move to the ERGIC and interact with the N protein to form MHV mature virions 16. The N- and C-terminal domains of the N protein interact with the viral RNA-packaging signal containing a 45-nucleotide stable stem-loop substructure to mediate packaging of the viral genomic RNA into MERS virus-like particles 17. The N protein also binds to the M protein in the ERGIC to facilitate virus assembly, which also involves the E protein 18, 19, although that interaction has not been well studied.

Severe acute respiratory syndrome coronavirus 2 (SARS-CoV-2) is the novel beta-coronavirus responsible for the coronavirus disease 2019 (COVID-19) pandemic. The World Health Organization reported on 17 January 2021 that a total of 93,194,922 people were infected with SARS-CoV-2 since January 2020, of which 2,014,729 had died (https://covid19.who.int). It was reported that SARS-CoV-2 positive cases have common symptoms such as anosmia, ageusia, loss of appetite, fever, muscle pain, fatigue/myalgia, dry cough, productive cough, diarrhea, difficulty in breathing, and sore throat 20. SARS-CoV-2 was firstly isolated from bronchoalveolar lavage fluid 21, 22 and human airway epithelial cells 23 of severe pneumonia patients. The genome sequences of SARS-CoV-2 were confirmed to be about 79% identical to SARS-CoV. The RBD region of the SARS-CoV-2 S protein binds to angiotensin-converting enzyme 2 (ACE2) on host cells and transmembrane protease serine 2 (TMPRSS2) activity facilitates the viral entry into the host cells 24-26. CoV N protein can bind to viral RNA forming ribonucleoprotein (RNP) complex. The N protein is involved in viral RNA transcription and replication, packaging of the viral genome, interferon inhibition, actin reorganization, host cell cycle progression and apoptosis 27-30. As there is currently little information regarding the N protein of SARS-CoV-2, it is vital to determine if results obtained from other coronaviruses, such as MERS-CoV and SARS-CoV, can be applied to SARS-CoV-2.

Here, we aimed to obtain novel information regarding viral protein-protein interactions in MERS-CoV-infected or SARS-CoV-2-infected cells and investigated whether the interactions can be used as a therapeutic target against these viruses.

## Materials and Methods

### Cell lines and viruses

Vero cells and Vero E6 cells, the African green monkey kidney cells, and Calu-3 cells, the human airway epithelial cells were purchased from the Korean Cell Line Bank (Seoul, Korea). The cells were cultured in Dulbecco's modified Eagle's medium (DMEM, Thermo Fisher Scientific, Waltham, MA, USA) containing 10% fetal bovine serum (FBS, Thermo Fisher Scientific), 25 mM HEPES, 100 U/ml penicillin, and 100 μg/ml streptomycin in 95% atmospheric air and 5% CO_2_ at 37˚C. MERS-CoV/KOR/KNIH/002_05_2015 was obtained from the Korea Centers for Disease Control and Prevention (Permission No. 1-001-MER-IS-2015001). SARS-CoV-2 (NCCP No. 43326) was provided by the National Culture Collection for Pathogens (Osong, Korea). MERS-CoV and SARS-CoV-2 preparation and cell culture procedures were performed in biosafety level-3 conditions.

### Virus amplification and quantification

Vero cells (2 × 10^5^ cells/well in 6-well plate) were plated with DMEM media containing 10% FBS and cultured at 37 °C in CO_2_ incubator. After overnight cell culture, the cells were washed with PBS and then MERS-CoV or SARS-CoV-2 at multiplicity of infection (MOI) 0.01 in PBS were added into each well and then incubated for 1 h at 37 °C in CO_2_ incubator. After incubation, 2 ml of DMEM/F12 medium for MERS-CoV or DMEM medium containing 2% FBS for SARS-CoV-2 was added and incubated at 37 °C in CO_2_ incubator for 3 days. The cell culture supernatants were harvested and centrifuged at 2,000 rpm for 10 min at 4 °C to remove the cell debris. The amplified virus supernatants were quantified by plaque assay. The quantified viruses (5 × 10^6^ plaque forming units (pfu)/ml) were aliquoted in 400 µl per Eppendorf tubes and then stored at -70 °C.

### Plaque formation assay

Vero cells (6 × 10^5^ cells/well) and Vero E6 cells (7 × 10^5^ cells/well) were plated on six-well plates (Corning, NY, USA) and cultured overnight at 37 °C in CO_2_ incubator. Vero cells and Vero E6 cells were washed with PBS and infected with the MERS-CoV and SARS-CoV-2, respectively, after ten-fold serial dilution. After 1 h incubation, the supernatants were removed. For MERS-CoV, the plates were replenished with 3 ml DMEM/F12 medium (Thermo Fisher Scientific) containing 0.6% oxoid agar and incubated at 37 °C for 4 days. For SARS-CoV-2, overlay DMEM/F12 medium (Thermo Fisher Scientific) containing 0.6% oxoid agar, and N-*p*-Tosyl-L-phenylalanine chloromethyl ketone (TPCK, 1 µg/ml)-treated trypsin was placed over the cell monolayer and incubated at 37 °C for 72 h. Plates were stained with 0.1% crystal violet for 1 h to verify plaque formation.

### Peptides synthesis

The following C-terminal domain of S protein (Spike CD) peptides were derived from the MERS-CoV S protein sequence [MERS-CoV/KOR/KNIH/002_05_2015 (GI: 829021049)] to investigate the interaction between the S and N proteins: Spike CD-Full, ^1318^TGCGTNCMGKLKCNRCCDRYEEYDLEPHKVHVH^1353^; Spike CD-F, ^1318^TGCGTNCMGKLKCNRC^1333^; Spike CD-M, ^1327^KLKCNRCCDRYEEYDL^1343^; and Spike CD-B, ^1336^DRYEEYDLEPHKVHVH^1353^. In order to ensure that the peptides could penetrate host cells, each peptide was conjugated with nine D-arginine residues at the N-terminus (R-Spike CD-MERS-CoV) and/or biotin at the C-terminus (R-Spike CD-Biotin, Spike CD-Full-Biotin, Spike CD-F-Biotin, Spike CD-M-Biotin, and Spike CD-B-Biotin). A control peptide (CP-1) was also conjugated with nine D-arginine residues (R-CP-1, NH_2_-d-RRRRRRRRR-AQARRKNYGQLDIFP-COOH) 31. The Spike CD-SARS-CoV-2 peptide was synthesized according to the SARS-CoV-2 S protein sequence (QHD43416, ^1234^LCCMTSCCSCLKGCCSCGSCCKFDEDDSEPVLKGVKLHYT^1273^) and conjugated with nine D-arginine residues at the N-terminus (R-Spike CD-SARS-CoV-2). All peptides were synthesized by Anygen Co., LTD. (Gwang Ju, South Korea).

### Antibodies

Monoclonal antibodies against the MERS-CoV S protein (anti-MERS-CoV S mAb; 492-1G10E4E2 clone) 32 and M protein (anti-MERS-CoV M mAb; M158-2D6F11 clone) 33 were prepared from hybridoma cells established after immunization of BALB/c mice with each peptide epitope formulated with a CpG-DNA-liposome complex as previously described 34. Spike-492 (^492^TKPLKYSYINKCSRLLSDDRTEVPQ^516^) and MERS-M158 (^158^CDYDRLPNEVTVAKPNVLIALKMVK^182^) were used as B-cell epitope sequences for the MERS-CoV S protein [Spike glycoprotein universal sequence (GI: 510785803)] and M protein, respectively. Rabbit anti-MERS N protein polyclonal antibody (anti-MERS-CoV N Ab, Cat. No.40068-RP02), rabbit anti-SARS-CoV-2 Spike protein polyclonal antibody (anti-SARS-CoV-2 S Ab, Cat. No. 40592-T62) and mouse anti-SARS-CoV-2 N protein monoclonal antibody (anti-SARS-CoV-2 S mAb, Cat. No. 40143-MM05) were purchased from Sino Biological (Vienna, Austria). Anti-β-actin antibody was obtained from Sigma-Aldrich (St. Louis, MO, USA).

### MERS-CoV infection and co-immunoprecipitation

Vero cells were cultivated for 12 h at a density of 6 × 10^5^ cells/10-cm dish. The cells were inoculated with MERS-CoV (0.1 MOI) in phosphate-buffered saline (PBS) and then incubated for 1 h in a 5% CO_2_ incubator at 37 °C. After incubation, the supernatants were removed, and the cultures were replenished with DMEM containing 25 mM HEPES, 100 U/ml penicillin, and 100 μg/ml streptomycin. Three days after infection, the MERS-CoV-infected Vero cells were lysed for 30 min at 4 °C in cell-lysis buffer (10 mM HEPES, 150 mM NaCl, 5 mM EDTA, 100 mM NaF, 2 mM Na_3_VO_4_, protease inhibitor cocktail, and 10% NP-40). The cell lysates were centrifuged to remove the cell debris and then incubated with anti-MERS-CoV S mAb or anti-MERS-CoV M mAb for 3 h at 4 °C. Protein A beads (CaptivA^tm^ PriMAB 52% (v/v) slurry, REPLIGEN, Waltham, MA, USA) were added, and the immunocomplexes were collected by centrifugation. The immunocomplexes were resolved by 4-12% gradient SDS-PAGE (Bolt^tm^ 4-12% Bis-Tris Plus gel; Thermo Fisher Scientific) and stained with Coomassie brilliant blue G-250.

### Analysis of MERS-CoV S protein binding

After immunoprecipitation of MERS-CoV-infected cell lysates with anti-MERS-CoV S mAb, immunocomplexes were resolved by 4-12% gradient SDS-PAGE. The protein bands of interest were cut out of the gels and analyzed by Proteinworks Co (Seoul, South Korea). Briefly, the protein bands from the gel were digested with trypsin, and the resulting peptides were separated using a Poros reverse phase R2 column (PerSeptive Biosystems, Framingham, MA, USA). The separated peptides were examined using ESI-TOF MS/MS (4700 MALDI-TOF/TOF, Applied Biosystems, Thermo Fisher Scientific). The peptide sequences were analyzed using the database from the National Center for Biotechnology Information (NCBI, http://www.ncbi.nlm.nih.gov).

### Western blotting and immunoprecipitation

Lysates of uninfected Vero cells, MERS-CoV-infected cells and SARS-CoV-2-infected cells were prepared using cell-lysis buffer (20 mM Tris·HCl pH 8.0, 5 mM EDTA, 150 mM NaCl, 100 mM NaF, 2 mM Na_3_VO_4_, 1% NP-40), centrifuged at 14,000 rpm at 4 °C for 20 min, and then separated in 4-12% Bis-Tris gradient gel (Thermo Fisher Scientific). The separated proteins were transferred onto nitrocellulose membranes and incubated with anti-MERS-CoV S mAb, anti-MERS-CoV M mAb, anti-MERS-CoV N Ab, anti-SARS-CoV-2 S Ab, anti-SARS-CoV-2 N mAb, or anti-β-actin antibody overnight at 4 °C. Then, the membranes were incubated with a horseradish peroxidase-conjugated secondary antibody, and the immunoreactive bands were developed using an enhanced chemiluminescence reagent (Thermo Fisher Scientific). To determine the binding properties of each MERS-CoV protein and SARS-CoV-2 protein, co-immunoprecipitation analysis was performed with anti-MERS-CoV S mAb, anti-MERS-CoV M mAb, or anti-SARS-CoV-2 S Ab. The co-immunoprecipitated proteins were identified using western blotting with anti-MERS-CoV S mAb, anti-MERS-CoV M mAb, anti-MERS-CoV N Ab, anti-SARS-CoV-2 S Ab, or anti-SARS-CoV-2 N mAb.

### Analysis of the interaction between MERS-CoV Spike CD peptides and the N protein

Lysates of MERS-CoV-infected Vero cells were incubated for 2 h at 4 °C with Spike CD-Full-Biotin, Spike CD-F-Biotin, Spike CD-M-Biotin, or Spike CD-B-Biotin. After the incubation, Streptavidin agarose (Thermo Fisher Scientific) was added. The immunocomplexes were collected by centrifugation, resolved by 10% SDS-PAGE, and then analyzed by western blotting using anti-N Ab. The band density was analyzed using the Quantity One program (Bio-Rad, Hercules, CA, USA). To determine the major region of MERS-CoV Spike CD involved in the interaction with the N protein, lysates of MERS-CoV-infected cells were incubated at 4°C with Spike CD-F, Spike CD-M, or Spike CD-B. After 1 h of incubation, Spike CD-Biotin was added to each sample and incubated for 2 h at 4 °C. The interaction of Spike CD-Biotin with the MERS-CoV N protein was determined by immunoprecipitation with Streptavidin agarose.

### Analysis of the interaction between SARS-CoV-2 Spike CD and the N protein

To investigate the interaction between SARS-CoV-2 Spike CD and the N protein, lysates of SARS-CoV-2-infected Vero cells were incubated for 1 h at 4 °C with Spike CD-MERS-CoV peptide, Spike CD-SARS-CoV-2 peptide or R-CP-1 peptide. After the incubation, anti-SARS-CoV-2 S Ab was added to each lysate and incubated for 2 h at 4 °C. Protein A beads were added to each sample and incubated for 2 h at 4°C. The co-immunoprecipitated proteins were collected by centrifugation and then co-immunoprecipitation analysis was performed with anti-SARS-CoV-2 S Ab and anti-SARS-CoV-2 N mAb.

### Cell penetration by the MERS-CoV Spike CD peptide

Vero cells (5 × 10^4^) were seeded onto cover glasses on 12-well plates. After 24 h, the cells were replenished with DMEM/F12 medium and incubated with R-Spike CD-MERS-CoV-Biotin in a 5% CO_2_ incubator for 30 min at 37 °C. The cells were fixed with 4% paraformaldehyde and subsequently blocked and permeabilized with phosphate-buffered saline with Tween-20 (PBST) containing 1% bovine serum albumin (BSA). Alexa Fluor-488-conjugated Streptavidin (Jackson ImmunoResearch laboratories Inc.) was added, and the cultures were incubated for 1 h. Then, the samples were washed in PBST, and Hoechst 33258 (Thermo Fisher Scientific) was added to stain the nuclei. The slides were analyzed by confocal microscopy using a Carl Zeiss LSM710 microscope (Carl Zeiss Co. Ltd. Oberkochen, Germany).

### Viability assays to determine the cytotoxic effect of the cell-penetrating peptides in cells

Vero cells (1 × 10^3^ cells/well) and Calu-3 cells (4 × 10^3^ cells/well) were cultured on 96-well plates in DMEM containing 2% FBS for 12 h. Then, the cells were incubated with R-Spike CD-MERS-CoV peptide, R-Spike CD-SARS-CoV-2 peptide, or R-CP-1 at the indicated concentrations for 3 days. The cells were then treated with 10 µl Cell Counting Kit-8 (CCK-8) solution (Dojindo Molecular Technologies, Rockville, MD, USA) for 2 h at 37 °C. Soluble formazan was measured by absorbance at 450 nm using a microplate reader (Thermo Fisher Scientific, Ratastie, Finland).

### Analysis of MERS-CoV S protein, SARS-CoV-2 S and N protein expression using confocal microscopy

Vero cells (5 × 10^4^ cells/well) and Calu-3 cells (5 × 10^4^ cells/well) were cultured overnight on cover glass on 12-well plates and then infected with MERS-CoV or SARS-CoV-2 (0.1 MOI) in PBS for 1 h at 37°C. After infection, the plates were replenished with DMEM/F12 medium (for MERS-CoV) or DMEM containing 2% FBS (for SARS-CoV-2) and then the cells were treated with cell-penetrating peptides (2 µM) at 6 h after each virus infection. After 48 h, the cells were fixed and then blocked. The permeabilized cells were incubated with anti-MERS-CoV S mAb, anti-SARS-CoV-2 S Ab, or anti-SARS-CoV-2 N mAb for 2 h. The cells were then washed with PBST containing 1% BSA and incubated with Alexa Fluor 488-conjugated secondary antibody (Thermo Fisher Scientific) for 1 h. The nuclei were stained with Hoechst 33258. The slides were examined using a Carl Zeiss LSM710 microscope.

### Inhibitory activity of the Spike CD-MERS-CoV peptide on MERS-CoV replication

Vero cells (6 × 10^5^ cells/well) were plated on six-well plates and cultivated for 12 h. MERS-CoV (200 pfu) was mixed with serially (two-fold) diluted R-Spike CD-MERS-CoV or R-CP-1 peptides in PBS. The mixtures were then added to Vero cells and incubated for 1 h at 37 °C. After incubation, the supernatants were removed, and plaque fomation assay was performed. Plaques were counted and compared between the peptide-treated samples and control samples treated with virus only.

### Inhibitory activity of the Spike CD-SARS-CoV-2 peptide on SARS-CoV-2 replication

Vero cells (5 × 10^4^ cells/well) or Calu-3 cells (5 × 10^4^ cells/well) were plated on 12-well plates and cultivated for 12 h. The cells were then infected with SARS-CoV-2 (0.1 MOI) in PBS for 1 h at 37°C. After infection, the plates were replenished with 1 ml DMEM containing 2% FBS. To investigate whether the R-Spike CD-MERS-CoV peptide, R-Spike CD-SARS-CoV-2 peptide, or R-CP-1 peptide could inhibit SARS-CoV-2 production in Vero cells and Calu-3 cells, the cells were treated with cell-penetrating peptide (2 µM) at 6 h after virus infection. Twenty-four hours after virus infection, the supernatants of the virus-infected cell cultures were collected, and the virus replication was quantified using real-time reverse transcription PCR (qRT-PCR) and plaque formation assay.

### Quantitative real-time RT-PCR

Viral RNAs were isolated from the supernatants of virus-infected cell cultures using the QIAamp Viral RNA Mini Kit (Catalog No. 52904, Qiagen, Hilden, Germany) according to the manufacturer's instructions. cDNA was prepared using a Reverse Transcription System kit (Catalog No. A3500, Promega, Madison, WI, USA). The primer sequences to quantify the RNA-dependent RNA polymerase (*RdRP*) gene of SARS-CoV-2 were 35: forward primer, 5′-GTGAAATGGTCATGTGTGGCGG-3'; reverse primer, 5'-CAAATGTTAAAAACACTATTAGCATA-3'; and TaqMan^®^ Probe, 5'-FAM-CAGGTGGAACCTCATCAGGAGATGC-TAMRA-3'. The primers and the probe sequence were synthesized by Genotech (Daejeon, South Korea). Ten microliters of GoTaq^®^ Probe qPCR Master Mix (catalog No. A6101, Promega, Madison, WI, USA) was added to 10 µl reaction mixture containing 125 nM each of forward and reverse primers, 250 nM TaqMan^®^ Probe, and 1 μl cDNA solution. After pre-denaturation at 95°C for 5 min, 45 cycles of PCR reaction were performed at 95 °C for 15 s and 60 °C for 1 min using Rotor-Gene Q (Qiagen). Copy numbers of the *RdRP* gene in the samples were calculated using a standard curve obtained with the cDNA levels of the *RdRP* gene.

### Phylogenetic analysis

Amino acid sequences corresponding to the Spike CDs and N proteins of betacoronaviruses were collected using the BLASTP program from NCBI (https://blast.ncbi.nlm.nih.gov/Blast.cgi?PROGRAM=blastp&PAGE_TYPE=BlastSearch&LINK_LOC=blasthome). The amino acid sequences were aligned using the Clustal W algorithm (Lasergene program version 5, DNASTAR Inc. Madison, WI). Phylogenetic trees of the betacoronaviruses were inferred using Dayhoff (for S protein) and JTT+G (for N protein) models of evolution (MEGA 7.0) 36. Support for the topologies was assessed by bootstrapping with 1,000 iterations. The accession number of amino acids used in this study was described in the Table S1.

### Statistical analysis

Results are shown as the mean ± standard deviation. Differences between two samples were evaluated using the Student's t-test and *p*-values < 0.05 were considered statistically significant.

## Results

### Interaction between the S protein and N protein of MERS-CoV

**Figure 1:**
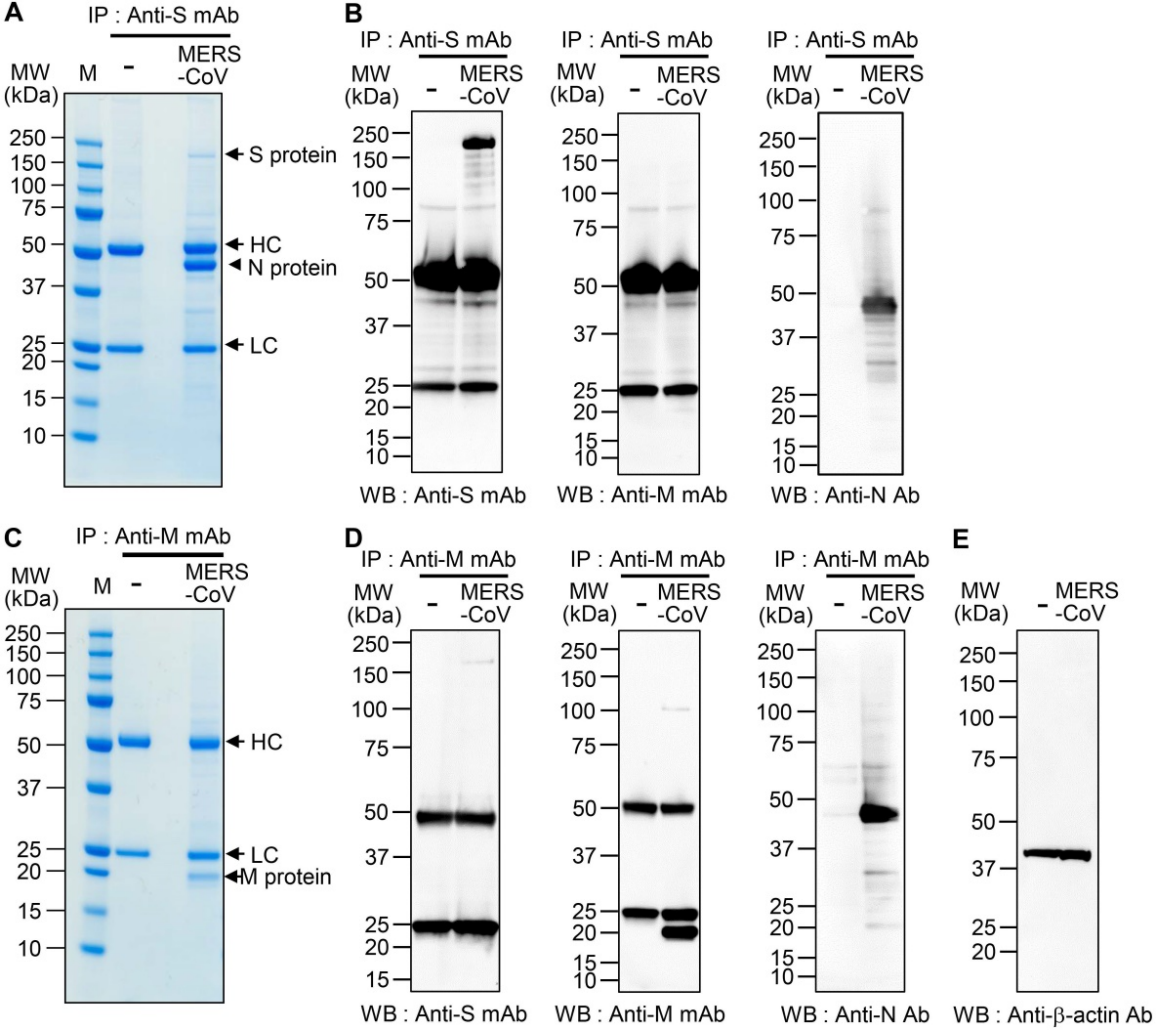
** Interaction of MERS-CoV S and M proteins with MERS-CoV N protein. (A and C)** Identification of proteins that bind S protein (A) and M protein (C). Lysates of uninfected and MERS-CoV (0.1 MOI)-infected Vero cells were prepared. The lysates (150 μg protein) were immunoprecipitated with anti-MERS-CoV S mAb (A) or anti-MERS-CoV M mAb (C), resolved by 4-12% gradient SDS-PAGE, and stained with Coomassie brilliant blue G-250. The indicated (arrowhead) protein band was digested with trypsin, and the digested peptides were analyzed by ESI-TOF MS/MS (A). HC, heavy chain. LC, light chain. **(B and D)** Association of S protein and M protein with N protein. Lysates of uninfected and MERS-CoV-infected Vero cells were prepared and immunoprecipitated with anti-MERS-CoV S mAb (B) or anti-MERS-CoV M mAb (D). (E) β-actin in the lysates from uninfected and MERS-CoV-infected Vero cells was used as a control. The immunocomplexes were subjected to western blotting with the indicated antibodies. The loading amount of immunoprecipitated sample used for the analysis of the N protein (anti-MERS-CoV N Ab) was half of that used for the analysis of the other proteins (anti-MERS-CoV S mAb, anti-MERS-CoV M mAb) (B and D). The exposure time for signal detection was 120 sec for anti-MERS-CoV S mAb and anti-MERS-CoV M mAb and 5 seconds for anti-MERS-CoV N Ab. Anti-S mAb, anti-MERS-CoV S mAb. Anti-M mAb, anti-MERS-CoV M mAb. Anti-N Ab, anti-MERS-CoV N Ab.

We performed immunoprecipitation with lysates of MERS-CoV-infected Vero cells and monoclonal antibodies against the MERS-CoV S protein (anti-MERS-CoV S mAb; 492-1G10E4E2 clone) 32 and M protein (anti-MERS-CoV M mAb; M158-2D6F11 clone) 33. Sodium dodecyl sulfate-polyacrylamide gel electrophoresis (SDS-PAGE) revealed an S-interacting protein with a molecular weight of ~ 45 kDa [Figure 1A (arrowhead)] but no prominent M-interacting proteins (Figure 1C). We fragmented the S-interacting protein in the gel with trypsin and analyzed the peptide fragments by electrospray ionization time-of-flight mass spectrometry/mass spectrometry (ESI-TOF MS/MS). The results revealed 19 peptide fragments with amino acid sequences matching 57.14% of the amino acids of the MERS-CoV N protein (Figure S1). Those results suggested that the S protein interacts strongly with the N protein in the MERS-CoV-infected cells. When we monitored the expression of the S, M, and N proteins in MERS-CoV-infected cells for 72 h by western blotting, we found that the N protein expression increased more rapidly and robustly than M or S protein expression after the onset of MERS-CoV infection (Figure S2).

To confirm the interaction between the S and N proteins in MERS-CoV-infected cells, we performed immunoprecipitation with anti-MERS-CoV S mAb or anti-MERS-CoV M mAb followed by western blot analysis with immunostaining with anti-MERS-CoV S mAb, anti-MERS-CoV M mAb, or anti-MERS-CoV N mAb. We detected N protein in the immunocomplex precipitated with anti-MERS-CoV S mAb (Figure 1B), which is in agreement with the MS analysis. However, we did not find any M protein in the immunocomplexes precipitated with anti-MERS-CoV S mAb. We also detected N protein in the immunocomplex precipitated with anti-MERS-CoV M mAb (Figure 1D-E), which is in agreement with previous reports on cells infected with SARS-CoV and MHV 16, 37. In addition, we detected a small amount of S protein in the immunocomplex precipitated with anti-MERS-CoV M mAb. We performed immunoprecipitation with anti-MERS-CoV N mAb and subsequent western blotting with anti-MERS-CoV S mAb or anti-MERS-CoV N Ab immunostaining; however, we could not verify the presence of the S and M proteins in the immunocomplexes because of the extremely strong intensity of the N protein band (data not shown).

### Interaction between Spike CD and the N protein of MERS-CoV

**Figure 2:**
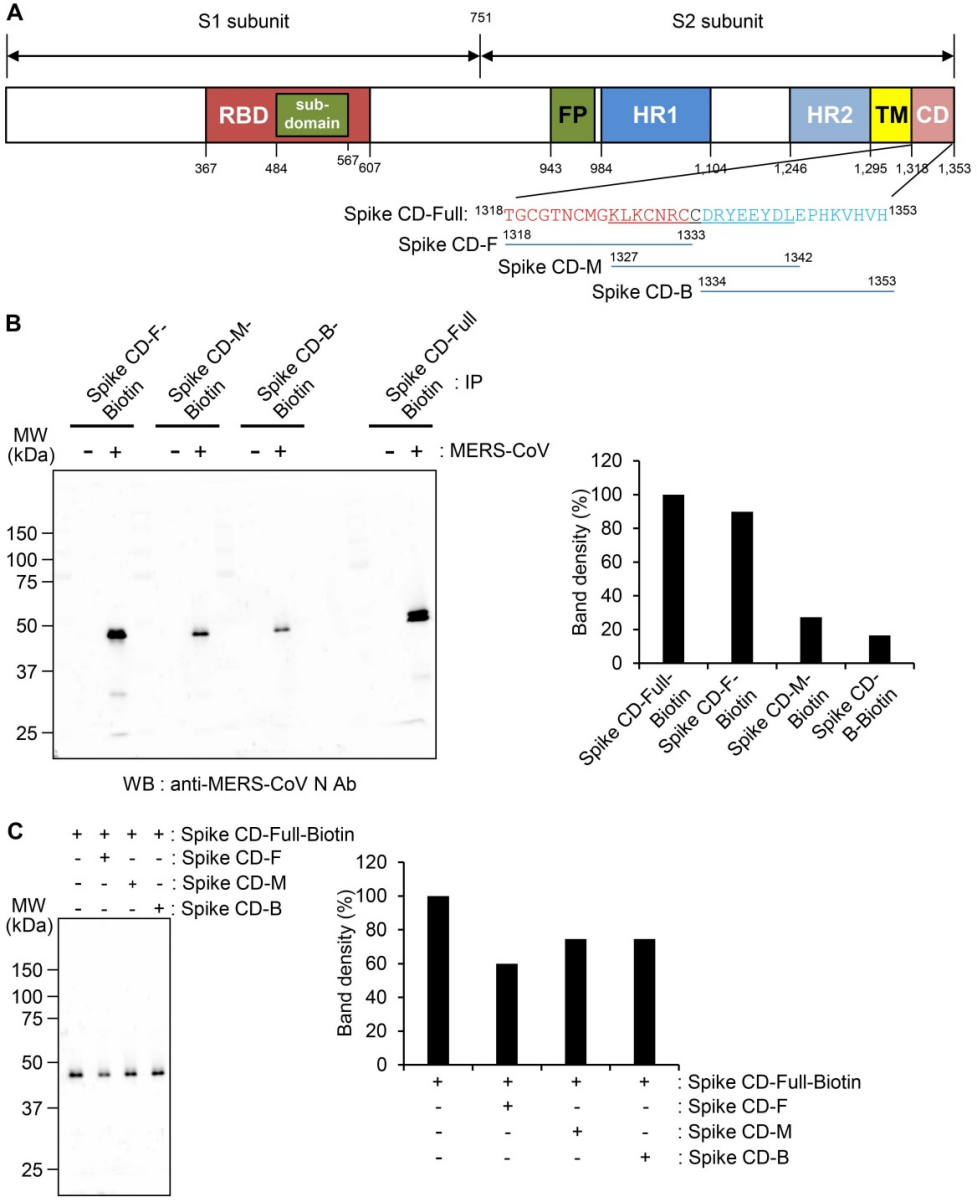
** Interaction of the cytoplasmic domain of MERS-CoV S protein with MERS-CoV N protein. (A)** Schematic diagram of the MERS-CoV S protein and the sequences of the cytoplasmic domain. RBD, receptor-binding domain; FP, fusion peptide; HR1 and HR2, heptad repeat regions 1 and 2; TM, transmembrane; CD, C-terminal domain; Spike CD, C-terminal domain of the S protein. Spike CD-Full, Spike CD-F, Spike CD-M, and Spike CD-B denote the synthetic peptide sequences. **(B)** Immunoprecipitation analysis. Lysates were prepared from uninfected and MERS-CoV-infected Vero cells. Immunocomplexes obtained using each biotinylated synthetic peptide were subjected to western blotting with anti-MERS-CoV N Ab. The right column represents the relative band intensities of the N protein. **(C)** Competition between Spike CD peptides and MERS-CoV Spike CD for interaction with MERS-CoV N protein. Cell lysates were prepared from MERS-CoV-infected Vero cells. The lysates were incubated with Spike CD-F, Spike CD-M, or Spike CD-B peptide for 2 h at 37 °C and then with biotinylated Spike CD-Full peptide (Spike CD-Full-Biotin) for 2 h at 37 °C. Immunocomplexes from Streptavidin beads were subjected to western blotting analysis with anti-MERS-CoV N protein antibody. The right column represents relative band intensities of the N protein.

We investigated the specificity of the interaction between the S and N proteins of MERS-CoV in detail. Because Spike CD might bind with N protein both in cells and in the assembled MERS-CoV, we synthesized four different peptides to study the interaction: full Spike CD (Spike CD-Full), front region of Spike CD (Spike CD-F), middle region of Spike CD (Spike CD-M), and back region of Spike CD (Spike CD-B; Figure 2A). We mixed biotinylated versions of each Spike CD peptide (Spike CD-Full-Biotin, Spike CD-F-Biotin, Spike CD-M-Biotin, and Spike CD-B-Biotin) with lysates of MERS-CoV-infected cells and confirmed the binding of the Spike CD-MERS-CoV peptides with N protein by immunocomplex analysis using Streptavidin beads. All of the Spike CD-MERS-CoV peptides interacted with the N protein to some degree; however, the Spike CD-Full and Spike CD-F peptide, which both contain the transmembrane domain proximal region (Figure 2B), displayed the strongest binding with the N protein.

To further investigate the region of the MERS-CoV Spike CD that interacts with the N protein, we analyzed the interaction of Spike CD-Full-Biotin with N protein in lysates of MERS-CoV-infected cells that had been pretreated with Spike CD-F, Spike CD-M, or Spike CD-B. Pretreatment with each of the peptides inhibited the interaction between Spike CD-Full and N protein by 30-40%, with Spike CD-F providing the strongest inhibition (Figure 2C). The small inhibitory effects can be explained by the fact that the N protein in the cell lysates was already bound to interacting proteins, including virus-derived S protein. Taken together, the results indicated that Spike CD-F includes the most important region of the Spike CD for interaction with the N protein, although Spike CD-Full is still the best peptide to use for studying the interaction between the S and N proteins.

### Host-cell penetration by Spike CD-MERS-CoV peptide inhibits MERS-CoV replication

**Figure 3:**
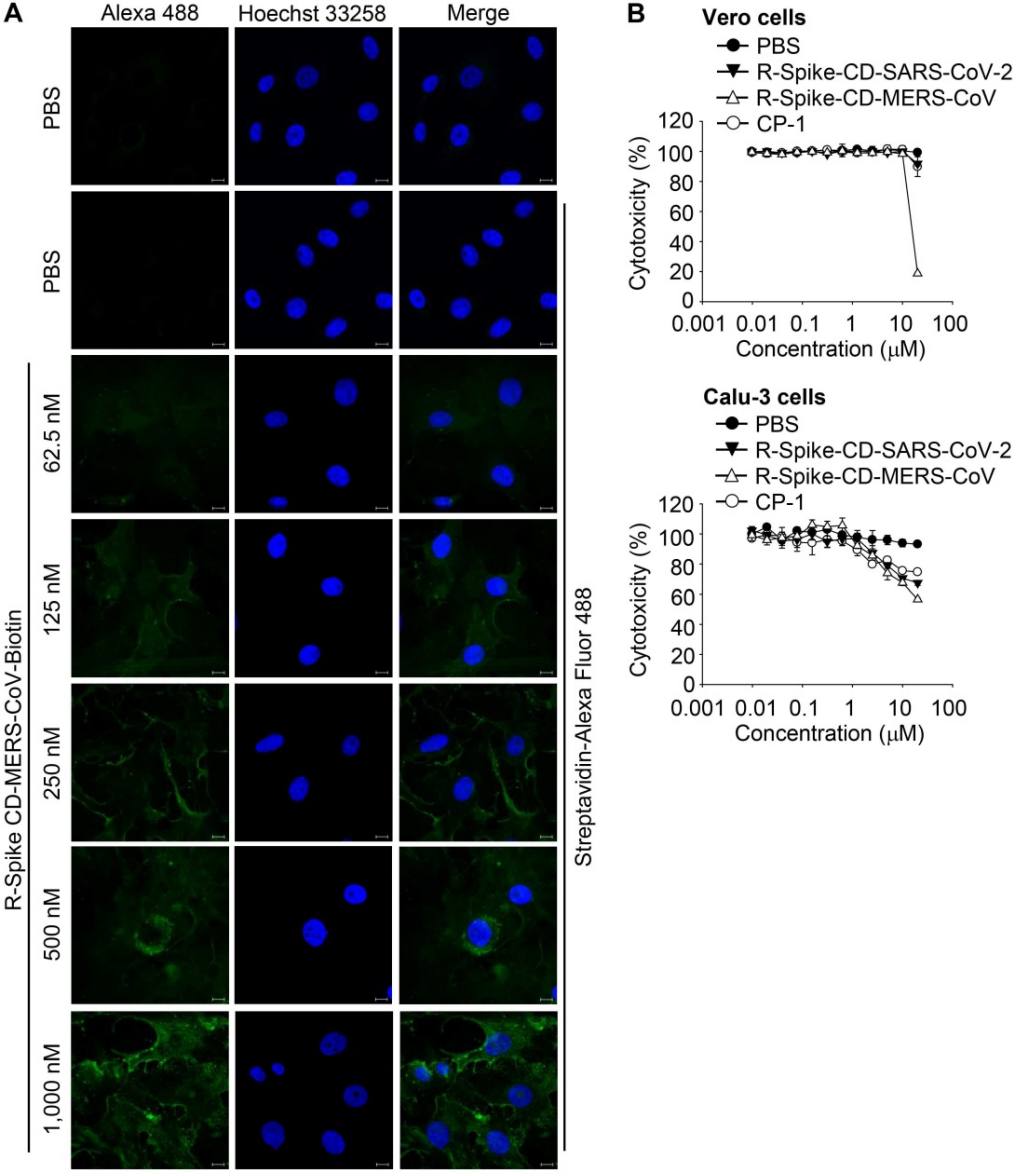
** Localization of MERS-CoV R-Spike CD in Vero cells and cytotoxicity of the cell-penetrating peptides. (A)** Vero cells were cultured for 24 h and then incubated with R-Spike CD-MERS-CoV-Biotin peptide for 30 min in a 5% CO_2_ incubator at 37 °C. The samples were fixed with 4% paraformaldehyde and permeabilized with 0.1% triton X-100. Cell-penetrated R-Spike CD-MERS-CoV-Biotin peptide was detected using Alexa Fluor-488-conjugated Streptavidin (Green) and a Carl Zeiss LSM710 microscope. Nuclei were stained with Hoechst 33258 (Blue). Scale bar, 10 µm. **(B)** Effect of cell-penetrating peptides on the growth of Vero cells and Calu-3 cells. Vero cells or Calu-3 cells were cultured with the indicated concentrations of cell-penetrating peptides for 3 days. The cells were incubated with CCK-8 solution, and then, soluble formazan was measured using a microplate reader. R-Spike CD-MERS-CoV, the peptide corresponding to the C-terminal domain of the MERS-CoV S protein conjugated with nine D-arginine residues at the N-terminus; R-Spike CD-MERS-CoV-Biotin, a biotinylated R-Spike CD-MERS-CoV peptide; R-Spike CD-SARS-CoV-2, the peptide corresponding to the C-terminal domain of the SARS-CoV-2 S protein conjugated with nine D-arginine residues at the N-terminus; R-CP-1, a nine D-arginine-conjugated control peptide.

To determine whether the Spike CD-Full peptide can inhibit the interaction between the viral S and N proteins and thus interfere with the intracellular production of MERS-CoV, we synthesized a Spike CD-Full peptide conjugated with nine D-arginine residues (R-Spike CD-MERS-CoV). We also synthesized a derivative with a C-terminal biotin (R-Spike CD-MERS-CoV-Biotin). We then treated Vero cells with R-Spike CD-MERS-CoV-Biotin and monitored uptake of the peptide using confocal microscopy. The confocal images showed that the fluorescence intensity increased in the cytoplasm in a dose-dependent manner (Figure 3A). To exclude potential side effects of cell-penetrating Spike CD peptides, we measured the cytotoxicity of R-Spike CD-MERS-CoV, R-Spike CD-SARS-CoV-2, and the control cell-penetrating peptide (R-CP-1) in Vero cells and Calu-3 cells. None of the cell-penetrating peptides induced cytotoxic effects at concentrations below 2 μM (Figure 3B).

**Figure 4:**
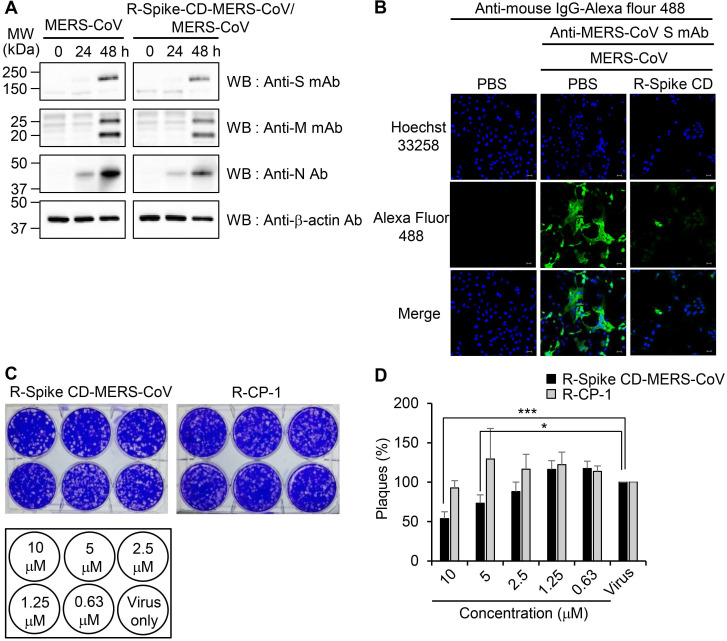
** Effects of R-Spike CD-MERS-CoV on MERS-CoV production. (A and B)** Reduction of MERS-CoV protein production by R-Spike CD-MERS-CoV. **(A)** Cell lysates were prepared at the indicated time points from Vero cells infected with MERS-CoV (0.1 MOI, with or without R-Spike CD-MERS-CoV). The cell lysates were analyzed by western blotting with the indicated antibodies. **(B)** Vero cells were infected with MERS-CoV (0.1 MOI, with or without R-Spike CD peptide-MERS-CoV) in serum-free medium. The cells were cultured for 48 h and then analyzed by confocal microscopy after staining with anti-MERS-CoV S mAb and then, Alexa Fluor 488-conjugated goat anti-mouse IgG antibody. Scale bar, 20 μm. **(C and D)** Inhibition of MERS-CoV plaque formation by R-Spike CD-MERS-CoV. MERS-CoV was mixed with two-fold serially diluted R-Spike CD-MERS-CoV and R-CP-1 (n = 3). The MERS-CoV virus (200 pfu)-peptide mixture was added to Vero cells in a 5% CO_2_ incubator at 37˚C. After 1 h of incubation, the medium was removed, and the cultures were replenished with DMEM/F12 containing 0.6% oxoid agar. After 4 days of incubation, plaque formation was verified by staining with crystal violet. **(C)** A representative picture showing plaque formation. **(D)** Quantification of the plaques formed by MERS-CoV infection after treatment with each peptide at the indicated concnetrations. Plaque numbers obtained in control plates treated with MERS-CoV virus only were taken as 100%. R-Spike CD-MERS-CoV, the peptide corresponding to the C-terminal domain of the S protein conjugated with nine D-arginine residues at the N-terminus; R-CP-1, nine-D-arginine-conjugated control peptide. *p < 0.05, ***p < 0.001 compared to virus-only controls. These results are representative of two independent experiments.

To examine the effect of cell penetration by Spike CD-MERS-CoV peptide on the expression of the S, M, and N proteins in MERS-CoV-infected cells, we infected Vero cells with MERS-CoV (0.1 MOI) with or without R-Spike CD-MERS-CoV treatment. Western blots with the appropriate antibodies indicated that the expression levels of the S, M, and N proteins increased markedly at 48 h post-infection and were moderately reduced by R-Spike CD-MERS-CoV treatment (Figure 4A). Considering that the N protein plays multiple roles in viral RNA transcription, replication, and packaging of the viral genome 17, 27, interaction of S and N proteins may be required for optimal function of N protein. Consequently, reduction of interaction by R-Spike CD-MERS-CoV treatment may result in reduced viral protein expression. Confocal microscopy more clearly revealed that R-Spike CD-MERS-CoV strongly reduced S protein expression (Figure 4B). The difference in the strength of the effect of the R-Spike CD-MERS-CoV treatment was probably due to the higher sensitivity of the imaging assay compared with that of the western blot assay.

To investigate the effect of cell penetration by Spike CD-MERS-CoV peptide on the intracellular replication of MERS-CoV, we infected Vero cells with MERS-CoV in the presence of R-Spike CD-MERS-CoV. Treatment with R-Spike CD-MERS-CoV reduced plaque formation in a dose-dependent manner (Figure 4C-D). Specifically, the plaque formation was reduced about 50% by 10 μM of R-Spike CD-MERS-CoV. In contrast, treatment with the control R-CP-1 had no effect on plaque formation. Those results confirmed that inhibition of the interaction between the Spike CD and the N protein by a cell-permeable Spike CD-MERS-CoV peptide reduces MERS-CoV replication in cells.

### Phylogeographic analysis of Spike CDs and N proteins

**Figure 5:**
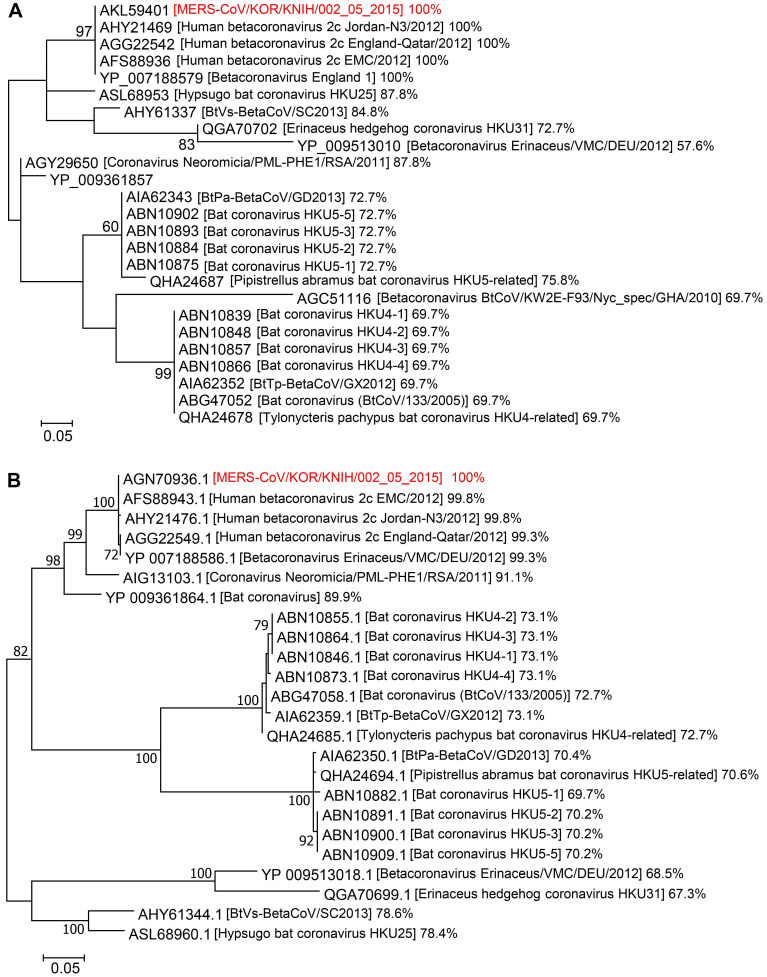
** Phylogenetic analysis of Spike CDs and N proteins of different betacoronavirus species. (A and B)** The phylogenetic trees of betacoronaviruses generated using Dayhoff (for Spike CDs) and JTT+G (for N proteins) models of evolution. Support for the topologies was assessed by bootstrap analysis with 1,000 iterations. The phylogenetic positions of (A) Spike CDs and (B) N proteins are shown in relation to representative betacoronaviruses. Relative homology of Spike CDs and N proteins is shown with homology to MERS-CoV/KOR/KNIH/002_05_2015 control taken as 100%. Spike CDs, S protein C-terminal domains.

We aligned the amino acid sequences of Spike CDs and N proteins from related betacoronaviruses to investigate their phylogenetic relationships. The Spike CD sequences of 24 strains of betacoronavirus lineage C had over 57% homology to that of MERS-CoV/KOR/KNIH/002_05_2015, the strain used in our study. There was 100% sequence homology between the Spike CD sequences of MERS-CoV strains from Korea (MERS-CoV/KOR/KNIH/002_05_2015), England (England 1), Qatar (England-Qatar/2012), Netherlands (Erasmus Medical Center/2012), and Jordan (Jordan-N3/2012). In addition, the Spike CD sequences of the MERS-CoV strains showed about 70% homology to that of bat betacoronavirus lineage C (Figure 5A). The N protein sequences showed over 67% homology among 23 strains of betacoronavirus lineage C. The N protein sequences of MERS-CoV strains from several countries shared 99% sequence homology with one another and also showed 70% homology to that of bat coronaviruses lineage C (Figure 5B). There was also high homology among the Spike CD and N protein sequences of betacoronavirus linage B species, including SARS-CoV and SARS-CoV-2. For example, the Spike CD sequences of SARS-CoV (^1216^LCCMTSCCSCLKG**A**CSCGSCCKFDEDDSEPVLKGVKLHYT^1255^) and SARS-CoV-2 (^1234^LCCMTSCCSCLKG**C**CSCGSCCKFDEDDSEPVLKGVKLHYT^1273^) were identical except for a single amino acid. By contrast, other betacoronavirus lineages showed low homology between their Spike CDs and N proteins. For example, the Spike CD sequence of MERS-CoV has 55% homology with the sequence of SARS-CoV-2.

### Cell penetration by Spike CD-SARS-CoV-2 peptide inhibits SARS-CoV-2 replication

**Figure 6:**
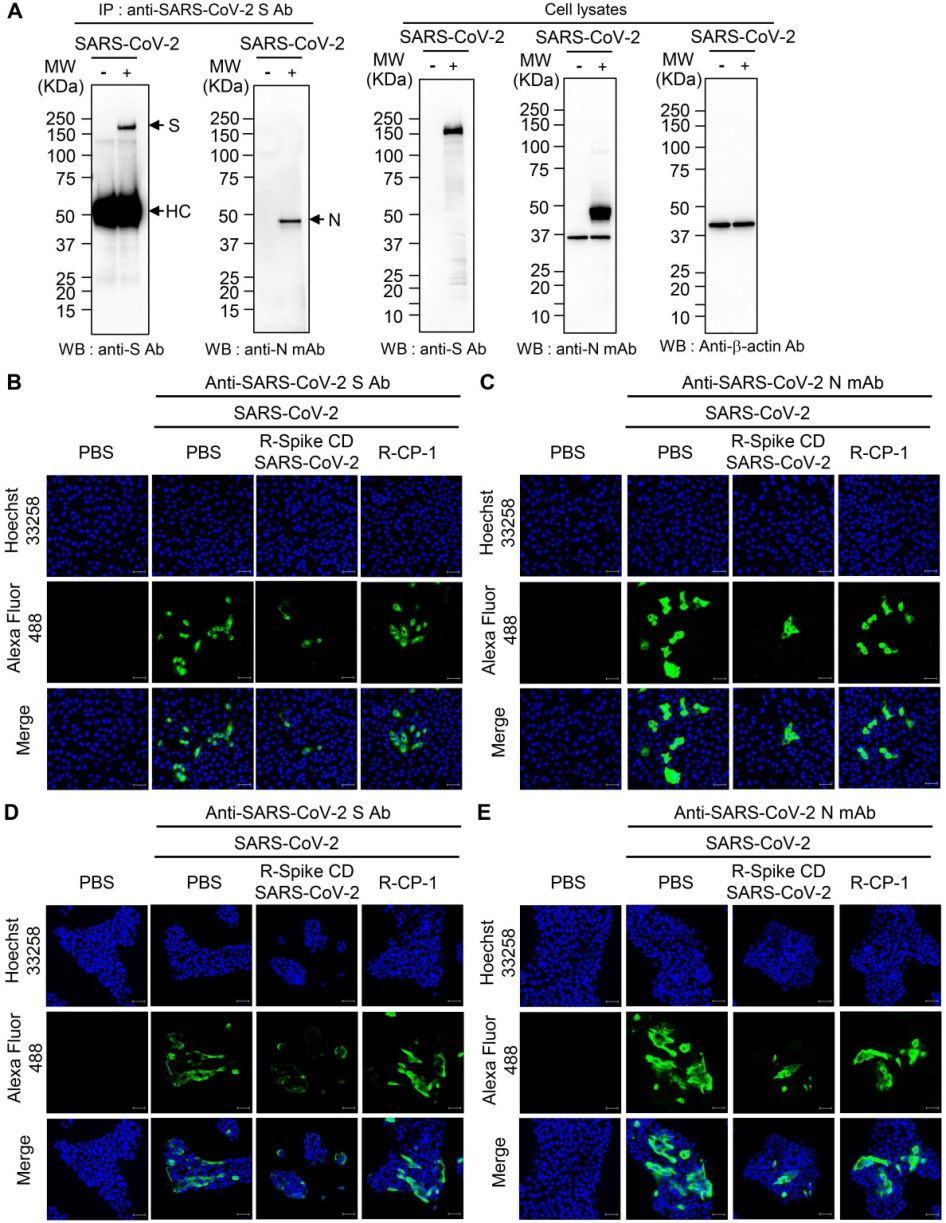
** Interaction of SARS-CoV-2 S protein with N protein and effects of R-Spike CD-SARS-CoV-2 on production of SARS-CoV-2 proteins. (A)** Interaction of SARS-CoV-2 S protein with N protein. Lysates were prepared from uninfected and SARS-CoV-2 (0.1 MOI)-infected Vero cells. The lysates were immunoprecipitated with anti-SARS-CoV-2 S mAb (left). The immunocomplexes were subjected to western blotting with anti-SARS-CoV-2 S mAb or anti-SARS-CoV-2 N Ab. The cell lysates were analyzed by western blotting with the indicated antibodies (right). Anti-S Ab, anti-SARS-CoV-2 S Ab. Anti-N mAb, anti-SARS-CoV-2 N mAb. **(B-E)** Effects of R-Spike CD-SARS-CoV-2 on production of SARS-CoV-2 proteins. Vero cells (B and C) and Calu-3 cells (D and E) were infected with SARS-CoV-2 (0.1 MOI) and then treated with PBS or 2 μM of cell-penetrating peptides (R-Spike CD-SARS-CoV-2 or R-CP-1) at 6 h after virus infection (n = 3) in DMEM medium containing 2% FBS. The cells were cultured for 48 h and then analyzed by confocal microscopy after staining with anti-SARS-CoV-2 S Ab (B and D) or anti-SARS-CoV-2 N mAb (C and E) and then, Alexa Fluor 488-conjugated secondary antibody. Scale bar, 20 μm. These results are representative of two independent experiments.

To examine the interaction between the S and N proteins in SARS-CoV-2-infected cells, we performed immunoprecipitation with anti-SARS-CoV-2 S mAb followed by western blot analysis with anti-SARS-CoV-2 S Ab, or anti-SARS-CoV-2 N mAb. We detected N protein in the immunocomplex precipitated with anti-SARS-CoV-2 S mAb (Figure 6A). We also investigated effect of Spike CD of SARS-CoV-2 on the interaction between the S and N proteins of SARS-CoV-2. When we added the peptide to the lysates of SARS-CoV-2-infected Vero cells and performed co-immunoprecipitation assay, Spike CD-SARS-CoV-2 peptide inhibited the interaction between the S and N protein of SARS-CoV-2 (Figure S3). However, addition of Spike CD-MERS-CoV peptide to the cell lysates did not affect the interaction (Figure S3).

To investigate the effect of cell penetration by Spike CD-SARS-CoV-2 peptide on the expression of the S and N proteins in SARS-CoV-2-infected cells, we infected Vero cells and Calu-3 cells with SARS-CoV-2 (0.1 MOI) and treated with R-Spike CD-SARS-CoV-2 or control peptide R-CP-1. Confocal images showed that R-Spike CD-SARS-CoV-2 but not R-CP-1 strongly reduced S protein and N protein expression in Vero cells (Figure 6B-C) and Calu-3 cells (Figure 6D-E). Then we further verified the effects of cell penetration by Spike CD-MERS-CoV peptide. R-Spike CD-MERS-CoV peptide treatment did not show significant inhibitory effect on expression of the S and N proteins in SARS-CoV-2-infected Vero cells (Figure S4).

**Figure 7:**
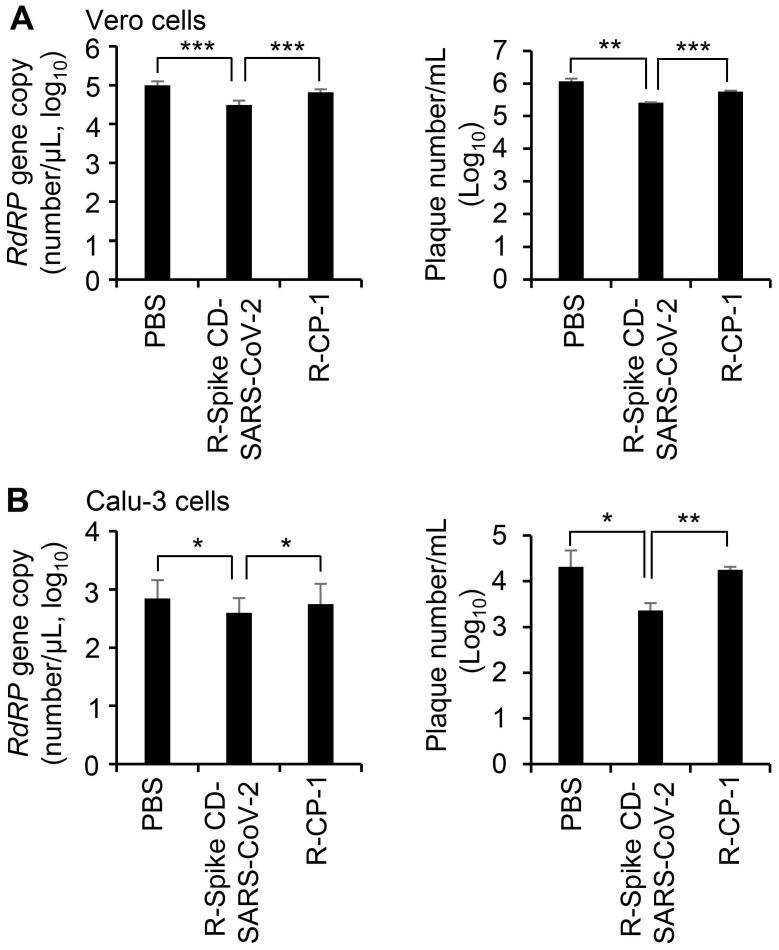
** Effect of R-Spike CD-SARS-CoV peptides on the replication of SARS-CoV-2. (A and B)** Vero cells (A) and Calu-3 cells (B) infected with SARS-CoV-2 (0.1 MOI) and then treated with PBS or 2 μM of cell-penetrating peptides (R-Spike CD-SARS-CoV-2 or R-CP-1) at 6 h after virus infection (n = 3). Supernatants of virus-infected cell cultures were collected at 24 h after virus infection. Virus replication was quantified by qRT-PCR analysis of the SARS-CoV-2 *RdRP* gene (left) and plaque formation assay (right). *p < 0.05, **p < 0.01, ***p < 0.001. These results are representative of two independent experiments.

To confirm the effect of cell penetration by Spike CD-SARS-CoV-2 peptide on the replication of SARS-CoV-2, we infected Vero cells (Figure 7A) and Calu-3 cells (Figure 7B) with SARS-CoV-2 (0.1 MOI) and treated with R-Spike CD-SARS-CoV-2 or control peptide R-CP-1. qRT-PCR analysis of the RNA-dependent RNA polymerase gene of SARS-CoV-2 and plaque formation assay revealed that replication was reduced in the presence of R-Spike CD-SARS-CoV-2 compared with that in control cells with no added peptides. R-CP-1 slightly reduced replication in Vero cells, suggesting nonspecific effects of the cell-permeable peptides (Figure 7). However, Spike CD peptide of MERS-CoV did not influence on SARS-CoV-2 replication (Figure S5). These results suggest that the degree of amino acid sequence identity between the Spike CD of a target virus and any given Spike CD peptide will determine the effectiveness of that peptide in reducing replication of the virus.

## Discussion

An understanding of the molecular interactions of viral proteins provides a basis for the development of therapeutics against virus infection. Until now, most studies have suggested that the interaction between the M and N proteins is the most important interaction for the assembly of coronaviruses such as MHV 16, 38 and SARS-CoV 17, 39. The M protein of MHV interacts with the N protein at the MHV budding site in the ERGIC, independently of viral RNA 38. In SARS-CoV, M proteins can self-assemble in an RNA-independent manner and are extracellularly released within membrane-enveloped vesicles 39. The C-terminal domain of the MHV M protein interacts with the C-terminal domain of the N protein to facilitate the incorporation of viral RNA and proteins into the viral envelope 16. In SARS-CoV, N-protein dimerization following the binding of viral RNA induced the interaction with M protein in the ERGIC 37. In the porcine deltacoronavirus, N-protein dimerization occurs at the N-terminus (N-finger motif) of the N protein, resulting in an increase in the viral RNA-binding affinity of the N protein 40. In addition to the M protein, the E protein is required for efficient assembly of coronavirus particles 15. MERS-CoV, a type of lineage C betacoronavirus, was only recently isolated, so its assembly process is not yet well known. Previously, it was reported that the S protein interacts indirectly with the N protein, possibly via the M protein, in MHV-infected cells 38. In this study, we observed a direct interaction between the C-terminal domain of S protein and the N protein in MERS-CoV-infected and SARS-CoV-2-infected cells. It is important to check whether this interaction is common in other coronaviruses. The detailed characteristics and function of the interaction also require further investigation.

The S protein of MERS-CoV has been investigated as a potential therapeutic target for the treatment of MERS-CoV infection 41. Many studies identified the RBD of the S protein as a target for therapeutic vaccines and reported the development of neutralizing antibodies against the RBD 32, 42-48. Peptides or inhibitors that interupt the interaction between the RBD and hDPP4 were also suggested 12, 49-51. However, no therapeutics have yet been approved for clinical application. We found that the cell-permeable peptide Spike CD suppressed virus production in MERS-CoV-infected cells, suggesting that the interaction between the S protein and N proteins is essential for MERS-CoV replication. Those results further suggest a novel approach to target the S protein for therapeutics against coronaviruses. It should be possible to rapidly screen for potent peptides or chemicals that inhibit the interaction between the S protein and the N protein *in vitro*.

Broad-spectrum antiviral strategies are needed to combat epidemic virus infections 52-54. To explore the spectrum of the antiviral activity of the Spike CD peptide, we performed a phylogenetic analysis of the amino acid sequences of the Spike CDs and N proteins of betacoronaviruses. The Spike CD and N protein of MERS-CoV showed high levels of sequence similarity to other lineage C betacoronaviruses, suggesting that cell-permeable derivatives of Spike CD with antiviral properties might be effective against a range of betacoronaviruses. Further identification of the exact region of the N protein that interacts with the Spike CD and comparative analysis of the homology of that region among different viral strains will be useful for the prediction of cross-reactivity among Spike CD peptides. The peptide-based antiviral strategy might help in the development of broad-spectrum antivirals that are effective against emerging novel coronaviruses. As soon as viral genome sequences become available, prompt analysis of the Spike CD sequences and evaluation of virus-specific Spike CD peptides might provide a timely defense against novel viruses. The Spike CD and N proteins of SARS-CoV-2 show high levels of sequence similarity to other lineage B betacoronaviruses. We created a Spike CD peptide based on the Spike CD sequence of SARS-CoV-2. When we treated SARS-CoV-2-infected cells with the cell-permeable Spike CD peptide, the peptide inhibited interaction between C-terminal domain of S protein and the N protein in SARS-CoV-2-infected cells. Our results also confirmed that the interaction between the S and N proteins is required for the replication of SARS-CoV-2 virions suggesting that the interaction can be a general and critical feature among coronaviruses. Further studies on other coronaviruses such as SARS-CoV and human coronaviruses are warranted to determine whether cell-permeable Spike CD peptides represent a broad-spectrum therapeutic approach to combat coronaviruses.

CoVs N protein consists of three parts containing two structural domains such as N-terminal domain and C-terminal domain, and three intrinsically disordered regions (IDRs) such as N-arm, central linker region (LKR) and C-tail. The N-terminal domains of CoVs N protein interact with 3' end of the viral RNA via electrostatic interactions for RNA binding. It has also been reported that the C-terminal domains are associated with RNA binding and oligomerization 27, 55. Three IDRs also contribute to RNA binding and oligomerization of the N protein 56. Therefore, many researchers investigated the structure of SARS-CoV-2 N protein for development of COVID-19 therapeutics targeting SARS-CoV-2 N protein to control N protein RNA-binding activity and N protein oligomerization 28, 30, 57. Recently determined crystal structures of the N-terminal domain 28 and C-terminal domain 30, 57 of SARS-CoV-2 N protein may support the understanding of the biological function of SARS-CoV-2 N protein and development of therapeutics. Based on the N protein structure of SARS-CoV-2, molecular docking studies 58 regarding N protein and Spike CD sequence of SARS-CoV-2 will be helpful to predict specific interaction sites and efficacy of specific Spike CD peptides. To investigate whether Spike CD of each coronavirus interacts with respective N protein and whether Spike CDs have cross-reactivity to N proteins of other coronaviruses, it is required to identify the region of N protein binding to Spike CD and to perform experiments such as interaction assays using prey & bait assay system 59. These approaches may contribute to development of broad-spectrum antiviral therapeutics.

Here, we suggest that interaction between the S and N proteins of MERS-CoV and SARS-CoV-2 can serve as a therapeutic target. However, the inhibitory peptides may have pitfalls such as poor permeability and low stability 60. Furthermore, cell penetrating peptides lack specificity of targeting cells and are susceptible to degradation during experimental or clinical procedures 61. Therefore, screenings of more stable inhibitors such as peptidomimetics, peptides with chemical modifications, or small molecule inhibitors are required to overcome these difficulties 60, 62. An efficient delivery system for the inhibitors will be also needed for therapeutic applications.

## Supplementary Material

Supplementary figures and tables.

## Abbreviations

COVID-19: coronavirus disease 2019
E protein: envelope protein
ERGIC: endoplasmic reticulum-Golgi intermediate compartment
IDR: intrinsically disordered region
LKR: linker region
M protein: membrane protein
MERS-CoV: Middle East respiratory syndrome coronavirus
N protein: nucleocapsid protein
PCR: polymerase chain reaction
qRT-PCR: quantitative real-time RT-PCR
RBD: receptor-binding domain
RdRP: RNA-dependent RNA polymerase
S protein: spike protein
SARS-CoV-2: severe acute respiratory syndrome coronavirus 2
Spike CD: C-terminal domain of the S protein
