# Supplementary material for: MERS-CoV and SARS-CoV-2 replication can be inhibited by targeting the interaction between the viral spike protein and the nucleocapsid protein
Source: Theranostics. 2021 Feb 6;11(8):3853–67. doi: 10.7150/thno.55647 (PMC7914343; doi:10.7150/thno.55647)
Supplement: Supplementary file 1 — Supplementary figures and tables. [file thnov11p3853s1.pdf]

Supplementary information

MERS-CoV and SARS-CoV-2 virus replication can be inhibited by targeting the interaction between the viral spike protein and the nucleocapsid protein

Byoung Kwon Park, Jinsoo Kim, Sangkyu Park, Dongbum Kim, Minyoung Kim, Kyeongbin Baek, Joon-Yong Bae, Man-Seong Park, Won-Keun Kim, Younghee Lee, Hyung-Joo Kwon

Figure S1

Figure S2

Figure S3

Figure S4

Figure S5

Table S1

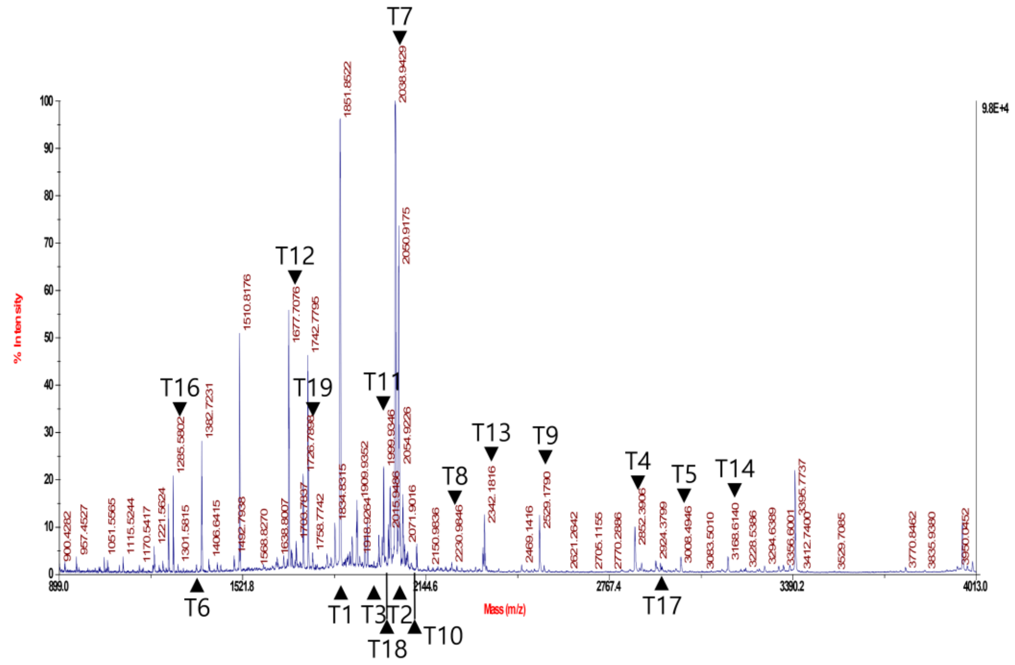

N protein [MERS-CoV]

Fragment	Region	Observed	Expected	Sequences
T1	9 - 25	1851.8967	1850.8894	R.AVSFADNNDITNTNLSR.G
T2	9 - 27	2064.9851	2063.9778	R.AVSFADNNDITNTNLSRGR.G
T3	35 - 52	1944.9475	1943.9490	R.AAPNNTVSWYTGLTQHGK.V
T4	53 - 79	2852.4336	2851.4263	K.VPLTFPPGQGVPLNANSTPAQNAGYWR.R
T5	53 - 80	3008.5459	3007.5386	K.VPLTFPPGQGVPLNANSTPAQNAGYWRR.Q
T6	85 - 97	1381.7649	1380.7576	K.INTGNGIKQLAPR.W
T7	98 - 114	2038.9905	2037.9832	R.WYFYYTGTGPEAALPFR.A
T8	98 - 117	2337.1724	2336.1651	R.WYFYYTGTGPEAALPFRAVK.D
T9	115 - 138	2529.1990	2528.1917	R.AVKDGIVWVHEDGATDAPSTFGTR.N
T10	139 - 158	2112.0432	2111.0359	R.NPNNDSAIVTQFAPGTKLPK.N
T11	156 - 174	2015.9979	2014.9906	K.LPKNFHIEGTGGNSQSSSR.A
T12	159 - 174	1677.7498	1676.7425	K.NFHIEGTGGNSQSSSR.A
T13	198 - 221	2342.2334	2341.2261	R.GTSPGPSGIGAVGGDLLYLDLLNR.L
T14	198 - 229	3168.6936	3167.6863	R.GTSPGPSGIGAVGGDLLYLDLLNRLQALESGK.V
T15	255 - 269	1686.8551	1685.8478	R.TSTKSFNMVQAFGLR.G
T16	259 - 269	1269.6205	1268.6132	K.SFNMVQAFGLR.G
T17	259 - 285	2923.4497	2922.4424	K.SFNMVQAFGLRGPGDLQGNFGDLQLNK.L
T18	396 - 413	1984.0023	1982.9950	R.TRTRPSVQPGPMIDVNTD.-
T19	398 - 413	1726.8332	1725.8260	R.TRPSVQPGPMIDVNTD.-

Figure S1. Identification of proteins binding the MERS-CoV S protein. The protein band that co-immunoprecipitated with the MERS-CoV S protein was digested in gel with trypsin. The resulting peptides were analyzed by ESI-TOF MS/MS. MS/MS analyses of the mass peaks (arrow) obtained from the ~45 kDa band revealed the peptide spectra of the MERS-CoV N protein.

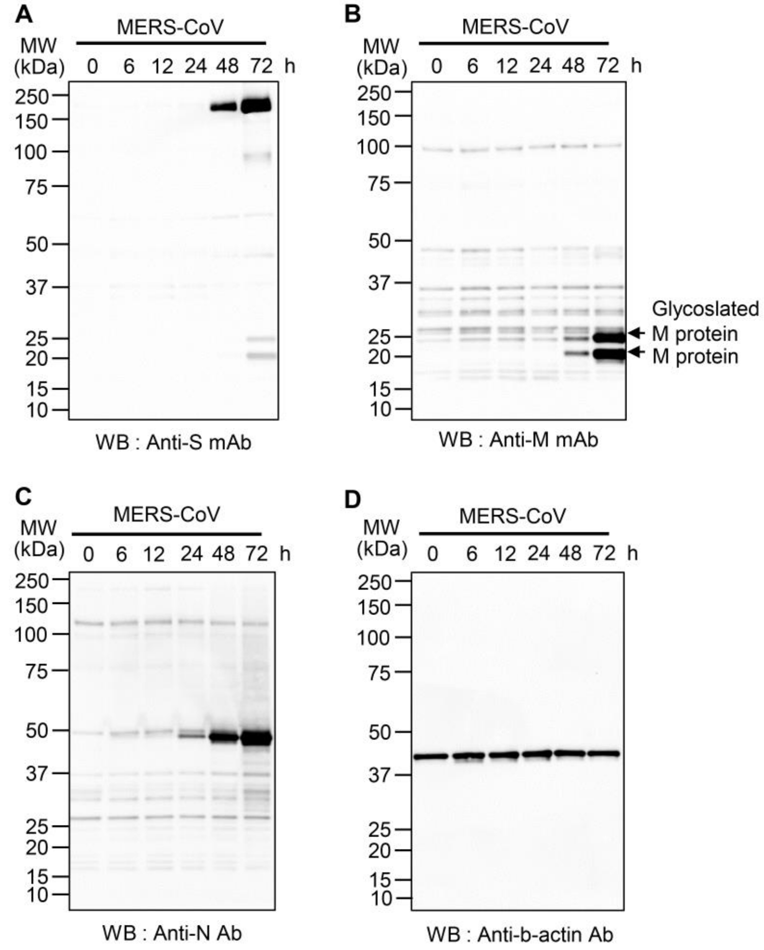

Figure S2. Expression of S, M, and N proteins in MERS-CoV-infected cells at 72 h after infection. Cell lysates were prepared from uninfected and MERS-CoV (0.1 MOI)-infected Vero cells. Cell lysates including 50 μg (**A, B, D**) and 25 μg (**C**) proteins were resolved by 4-12% gradient SDS-PAGE and analyzed by western blotting with the indicated antibodies. The exposure time for signal detection was 60 s (A, B, D) and 5 s (C).

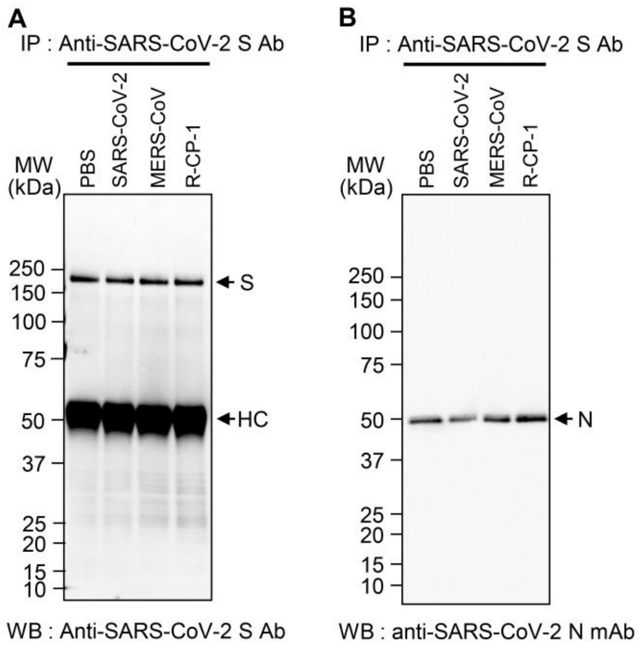

Figure S3. Interaction of SARS-CoV-2 Spike CD with N protein. Vero cells were infected with SARS-CoV-2 (0.1 MOI) for 72 h and then cell lysates were prepared. The cell lysates were mixed with Spike CD-SARS-CoV-2 peptide, Spike CD-MERS-CoV peptide, or R-CP-1 peptide (5 μg peptide/each reaction) and then incubated for 2 h at 4°C. Anti-SARS-CoV-2 S Ab was added to each lysate and then co-immunoprecipitated proteins were collected with Protein A bead. Co-immunoprecipitated samples were analyzed by Western blotting using anti-SARS-CoV-2 S Ab (**A**) and anti-SARS-CoV-2 N mAb (**B**). SARS-CoV-2, Spike CD-SARS-CoV-2 peptide; MERS-CoV, Spike CD-MERS-CoV peptide.

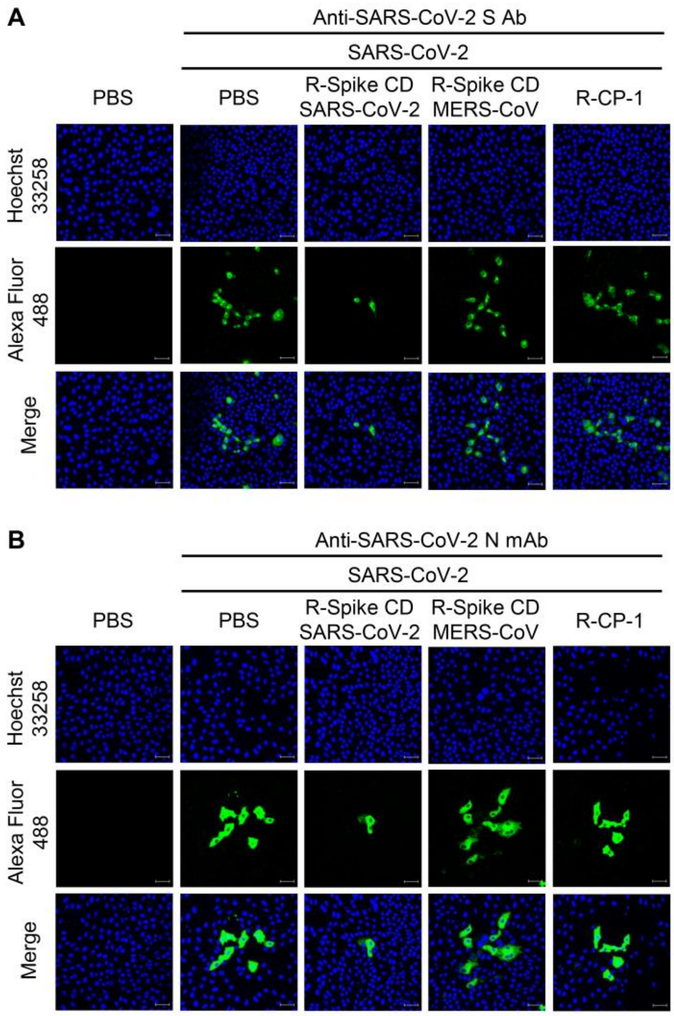

Figure S4. Effects of cell-penetrating Spike CD peptides of coronaviruses on SARS-CoV-2 protein production. Vero cells were infected with SARS-CoV-2 (0.1 MOI) and then treated with PBS or 2 μM of cell-penetrating peptides (R-Spike CD-SARS-CoV-2, R-Spike CD-MERS-CoV, or R-CP-1) at 6 h after virus infection (n = 3) in DMEM medium containing 2% FBS. The cells were cultured for 48 h and then analyzed by confocal microscopy after staining with anti-SARS-CoV-2 S Ab (**A**) or anti-SARS-CoV-2 N mAb (**B**) and then Alexa Fluor 488-conjugated secondary antibody. Scale bar, 20 μm.

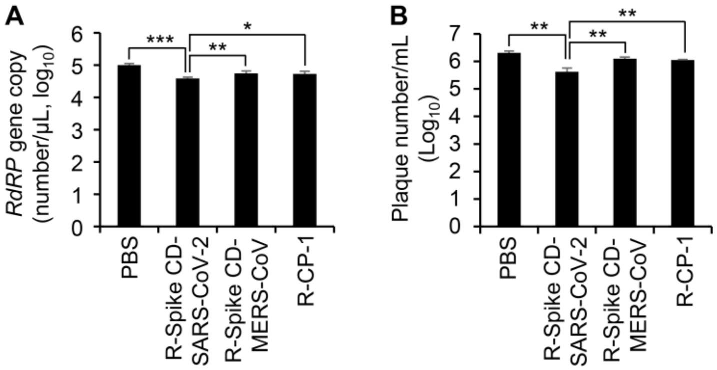

Figure S5. Effect of R-Spike CD-MERS-CoV peptide on the replication of SARS-CoV-2. Vero cells infected with SARS-CoV-2 (0.1 MOI) and then treated with PBS or 2 μM of cell-penetrating peptides (R-Spike CD-SARS-CoV-2, R-Spike CD-MERS-CoV, or R-CP-1) at 6 h after virus infection (n = 3). Supernatants of virus-infected cell cultures were collected at 24 h after virus infection. Virus replication was quantified by qRT-PCR analysis of the SARS-CoV-2 *RdRP* gene (**A**) and plaque formation assay (**B**). *$p < 0.05$, **$p < 0.01$, ***$p < 0.001$.

Table S1. Accession number of MERS-CoV S and N protein amino acids used in this study

Protein	Strain	Accession number
S protein		
	KOR-KNIH-002/05/2015	AKL59401
	Human betacoronavirus 2c Jordan-N3/2012	AHY21469
	Human betacoronavirus 2c England-Qatar/2012	AGG22542
	Human betacoronavirus 2c EMC/2012	AFS88936
	Betacoronavirus England 1	YP_007188579
	Hypsugo bat coronavirus HKU25	ASL68953
	BtVs-BetaCoV/SC2013	AHY61337
	Erinaceus hedgehog coronavirus HKU31	QGA70702
	Betacoronavirus Erinaceus/VMC/DEU/2012	YP_009513010
	Coronavirus Neoromicia/PML-PHE1/RSA/2011	AGY29650
	Betacoronavirus PREDICT/PDF-2180	YP_009361857
	BtPa-BetaCoV/GD2013	AIA62343
	Bat coronavirtus HKU5-5	ABN10902
	Bat coronavirtus HKU5-3	ABN10893
	Bat coronavirtus HKU5-2	ABN10884
	Bat coronavirtus HKU5-1	ABN10875
	Pipistrellus abramus bat coronavirus HKU5-related	QHA24687
	Betacoronavirus BtCoV/KW2E-F93/Nyc_spec/GHA/2010	AGC51116
	Bat coronavirtus HKU4-1	ABN10839
	Bat coronavirtus HKU4-2	ABN10848
	Bat coronavirtus HKU4-3	ABN10857
	Bat coronavirtus HKU4-4	ABN10866
	BtTp-BetaCoV/GX2012	AIA62352
	Bat coronavirtus (BtCoV/133/2005)	ABG47052
	Tylonycteris pachypus bat coronavirus HKU4-related	QHA24678
N protein		
	KOR-KNIH-002/05/2015	AGN70936.1
	Human betacoronavirus 2c EMC/2012	AFS88943.1
	Human betacoronavirus 2c Jordna-N3/2012	AHY21476.1
	Human betacoronavirus 2c England-Qatar/2012	AGG22549.1
	Betacoronavirus Erinaceus/VMC/DEU/2012	YP 007188586.1
	Coronavirus Neoromicia/PML-PHE1/RSA/2011	AIG13103.1
	Bat coronavirus	YP 009361864.1
	Bat coronavirus HKU4-2	ABN10855.1
	Bat coronavirus HKU4-3	ABN10864.1
	Bat coronavirus HKU4-1	ABN10846.1
	Bat coronavirus HKU4-4	ABN10873.1
	Bat coronavirus (BtCoV/133/2005)	ABG47058.1
	BtTp-BetaCoV/GX2012	AIA62359.1
	Tyonycteris pachypus bat coronavirus HKU4-related	QHA24685.1
	BtPa-BetaCoV/GD2013	AIA62350.1
	Pipistrellus abramus bat coronavirus HKU5-related	QHA24694.1
	Bat coronavirus HKU5-1	ABN10882.1
	Bat coronavirus HKU5-2	ABN10891.1
	Bat coronavirus HKU5-3	ABN10900.1
	Bat coronavirus HKU5-5	ABN10909.1

	Betacoronavirus Erinaceus/VMC/DEU/2012	YP 009513018.1
	Erinaceus hedgehog coronavirus HKU31	QGA70699.1
	BtVs-BetaCoV/SC2013	AHY61344.1
	Hypsugo bat coronavirus HKU25	ASL68960.1
